# Prevalence of chronic kidney disease in Thai adults: a national health survey

**DOI:** 10.1186/1471-2369-10-35

**Published:** 2009-10-31

**Authors:** Leena Ong-ajyooth, Kriengsak Vareesangthip, Panrasri Khonputsa, Wichai Aekplakorn

**Affiliations:** 1 Renal Division, Department of Medicine, Faculty of Medicine Siriraj Hospital, Mahidol University, Bangkok, Thailand; 2 Setting Priorities using Information on Cost-Effectiveness (SPICE) Project, Ministry of Public Health, Bangkok, Thailand; 3 Community Medicine Center, Faculty of Medicine, Ramathibodi Hospital, Mahidol University, Bangkok, Thailand

## Abstract

**Background:**

The prevalence of patients with end stage renal disease (ESRD) who need dialysis and/or transplantation has more than doubled in Thailand during the past two decades. It has been suggested that therapeutic strategies to reduce the risk of ESRD and other complications in CKD are now available, thus the early recognition and the institution of proven therapeutic strategies are important and beneficial. We, therefore, aimed to determine the prevalence of CKD in Thai adults from the National Health Examination Survey of 2004.

**Methods:**

Data from a nationally representative sample of 3,117 individuals aged 15 years and older was collected using questionnaires, physical examination and blood samples. Serum creatinine was measured by Jaffé method. GFR was estimated using the Chinese modified Modification of Diet in Renal Disease Study equation. Chronic kidney Disease (CKD) stages were classified based on Kidney Disease Outcome Quality Initiative (K/DOQI).

**Results:**

The prevalence of CKD in Thai adults weighted to the 2004 Thai population by stage was 8.1% for stage 3, 0.2% and 0.15% for stage 4 and 5 respectively. Compared to non-CKD, individuals with CKD were older, had a higher level of cholesterol, and higher blood pressure. Those with cardiovascular risk factors were more likely to have CKD (stage 3-5) than those without, including hypertension (OR 1.6, 95%CI 1.1, 3.4), diabetes (OR 1.87, 95%CI 1.0, 3.4). CKD was more common in northeast (OR 2.1, 95%CI 1.3, 3.3) compared to central region. Urinalysis was not performed, therefore, we could not have data on CKD stage 1 and 2. We have no specific GFR formula for Thai population.

**Conclusion:**

The identification of CKD patients should be evaluated and monitored for appropriate intervention for progression to kidney disease from this screening.

## Background

The prevalence of patients with end stage renal disease (ESRD) who need dialysis and/or transplantation has more than doubled in Thailand during the past two decades [1,2]. In addition to being at risk of ESRD, people with chronic kidney disease (CKD) have an increased risk of cardiovascular death [3,4]. Besides, chronic kidney disease is associated with an increased risk of a multitude of adverse health outcomes, including end stage kidney disease (ESRD) as well as a substantial reduction in life expectancy [3,5]. ESRD has a profound effect on morbidity, mortality and quality of life and imposes a substantial burden on health care expenditure [6]. Therapeutic strategies to reduce the risk of ESRD and other complications in CKD are now available [7]. So early recognition and the institution of proven therapeutic strategies are important and beneficial.

Large population representative surveys in the United States and Australia have reported the prevalence of CKD in these countries [8,9]. However, much less information is available on prevalence rates in developing regions [10]. Given their the limited ability to afford to provide dialysis and the rapidly increasing prevalence of diabetes and hypertension that may predispose to the development of CKD, preventive strategies are the only cost effective measure in these countries [11].

The purpose of this study is to estimate the prevalence of CKD among adults in Thailand.

## Methods

The third National Health Examination Survey (NHES III) is a national representative survey of Thai population and was conducted by the Health Research Institute. The study was approved by the Ethical Review Committee for Research in Human Subjects, Ministry of Public Health and informed consent was obtained from all participants. Data were collected between January and May of the year 2004.

### Sampling design

Multistage stratified cluster sampling technique was used to recruit study participants. The sampling technique was described elsewhere [12]. Briefly, the country was firstly divided into 13 areas (12 public health areas and Bangkok) using data from the national registration. Next, for each area, except Bangkok, three provinces were randomly selected using proportional to size (PPS) probability. Then each selected province was divided into rural and urban areas. The areas were subsequently sampled for 9 villages or electoral units using PPS. The last sampling step was to randomly select 15 people for 4 distinct age and sex groups, males aged 15-59 and males aged 60 years or above, and females aged 15-59 and females aged 60 years or above, using simple random sampling. Bangkok was divided into 6 administrative areas and 9 electoral units were selected for each area using PPS. The final sample size for NHES III was 39,290 compared to 42,120 planned (93.3%). Data were collected through interviews, population characteristics included demographic, medical history and treatment. Appointments were made with individuals in advance for blood sample collection. Venous blood samples were obtained by trained nurses under close supervision of physicians, using aseptic technique vacuum syringes and disposable needles. Physical examinations were performed and laboratory blood tests were obtained for measurement of fasting plasma glucose, and total cholesterol but not creatinine. However, serum samples were obtained and stored at -80 degree Celsius.

### Measurement of serum creatinine

The serum samples collected at time in the NHES III were stored at -80 degree Celsius. In the present study, a total of 3,120 serum samples were randomly selected proportional to the sample size of each age group, sex, urban/rural and public health area strata. In 2007, the selected frozen serum samples were thawed and analyzed for serum creatinine using a modified kinetic Jaffé method using automatic analyzer MERCK model MEGA, Germany under the External Quality Assurance Services (EQAS) of Biorad Laboratories, USA (Monthly Clinical Chemistry Quality Assessment Programme).

### Definitions

In this study, chronic kidney disease (CKD) means individuals who self-reported as receiving dialysis or who had glomerular filtration rates < 60 milliliters/minute (mL/min) calculated using the Modification of Diet in Renal Disease (MDRD) formula with adjustment for Asian ethnicity (GFR = 186 × serum creatinine^-1.154 ^× age (in years)^-0.203 ^× 1.233 (for Asian) × 0.742 (if female)). Hypertension means having systolic blood pressure (SBP) ≥140 mmHg or diastolic blood pressure (DBP) ≥90 mmHg or having used blood pressure lowering medication over the past 2 weeks.

The CKD staging was categorized based on the classification system established by the National Kidney Foundation Kidney Disease Outcomes Quality Initiatives. In the absence of urine albumin data, we only report stages 3 to 5. Stages 3 to 5 are defined as follows: stage 3, a GFR of 30 to 59 mL/min/1.73 m^2^; stage 4, a GFR of 15-29 mL/min/1.73 m^2^; stage 5, a GFR of less than 15 mL/min/1.73 m^2^.

An average of 2 measurements of blood pressure with the lowest variation of the differences between SBP and DBP was used. Diabetes mellitus means having fasting blood sugar ≥126 mg/dL or currently using medication or insulin for lowering blood sugar. Hypercholesterolemia means having total cholesterol ≥240 mg/dL (6.7 mmol/L) or currently using cholesterol lowering medications. Body mass index is the ratio between body mass (in kilogram) and the product of height (in metre). Smoking refers to those who had smoked 100 cigarettes or more and were using tobacco at the time of interview.

### Statistical analysis

Analyses with appropriate sample weights taking into account of the complex survey design were used to obtain unbiased estimates for prevalence of CKD and average measures of kidney function in Thai population. Estimates by demographic characteristic and risk factors were age-standardized to the 2004 Thai population.

Adjusted Wald tests were used to test for statistically significant difference between groups at 95% confidence level (*P *< 0.05). Logistic regression was used to measure the association between major risk factors and CRF (GFR < 60 mL/min). The independent variables were age, region, sex, BMI, total cholesterol, hypertension, diabetes mellitus, and smoking.

Stata 9.2 software program was used for all data analysis (Stata corporation, Texas).

## Results

Table 1 shows baseline characteristics of 3,117 participants, 1,557 male and 1,560 female by sex, age, FPG, total cholesterol, BMI, blood pressure, hypertension creatinine, and estimated GFR.

**Table 1:** Characteristic of sample in a subsample of Thai adults aged 15 years in NHES III, 2004

Factors	Men (%) n = 1557	Women (%) n = 1560	Total (%) n = 3117
Age (mean, SE)	33.8 ± 0.5	33.3 ± 0.5	33.6 ± 0.4
Sex (population)	49.1%	50.9%	100%
Age group (yr)			
15-29	390 (25.0%)	390 (25.0%)	780 (25.0%)
30-44	390 (25.0%)	390 (25.0%)	780 (25.0%)
45-59	388 (24.9%)	390 (25.0%)	778 (25.0%)
≥ 60	389 (25.0%)	390 (25.0%)	779 (25.0%)
Urban/rural			
Urban	839 (53.9%)	840 (53.9%)	1679 (53.9%)
Rural	718 (46.1%)	720 (46.1%)	1438 (46.1%)
Region			
Central	479 (30.8%)	480 (30.8%)	959 (30.8%)
Northeast	360 (23.1%)	360 (23.1%)	720 (23.1%)
North	360 (23.1%)	360 (23.1%)	720 (23.1%)
South	238 (15.3%)	240 (15.4%)	478 (15.3%)
Bangkok	120 (7.7%)	120 (7.7%)	240 (7.7%)
Hypertension* (%)	26.0	19.1	22.5
Diabetes* (%)	6.6	7.5	7.0
TC* (mean ± SE)	188.9 ± 2.0	196.3 ± 2.0	192.8 ± 1.8
High cholesterol* (TC ≥ 240 mg/dL)	12.8	16.5	14.7
BMI* (mean ± SE), kg/m^2^	22.6 ± 0.2	23.8 ± 0.2	23.2 ± 0.1
Smoking* (%)	46.0	2.6	24.3
Creatinine* (mean ± SE), mg/dL	1.3 ± 0.01	1.1 ± 0.01	1.2 ± 0.01
GFR* (mean ± SE), mL/min	82.7 ± 0.4	75.2 ± 0.3	78.9 ± 0.3

(*age standardized)

Mean systolic blood pressure was 115.3 mmHg (male 118.5 mmHg and female 112.2 mmHg), diastolic blood pressure, 74.7 mmHg (male 76.8 mmHg and female 72.8 mmHg). Prevalence of hypertension was 22.5% with higher prevalence in male than in female (25.9% vs 19.1%), FPG 92.1 mg/dL (male 93.9 mg/dL and female 90.5 mg/dL), with prevalence of diabetes 7.0%. Average BMI was higher in female than male: BMI 23.2 kg/m^2 ^(male 22.6 kg/m^2 ^and female 23.8 kg/m^2^). Mean creatinine level was greater in men than in women (1.3 mg/dL vs 1.1 mg/dL). The estimated mean GFR according to the modified MDRD was 78.9 mL/min (men 82.7 mL/min and women 75.2 mL/min).

### Prevalence of Chronic Kidney Disease (Table 2)

**Table 2:** Age-standardized prevalence (95% confidence interval) of CKD by sex, urban/rural and region using MDRD (× 1.233) formula

	Stage3	Stage4	Stage5	Total
**Total**	8.1 (7.3, 9.1)	0.2 (0.1, 0.5)	0.2 (0.1, 0.4)	8.9 (8.0, 9.9)
**Sex**				
Men	4.8 (3.8, 6.0)	0 (0, 0.4)	0.1 (0, 0.6)	5.5 (4.3, 6.8)
Women	11.4 (10.1, 12.9)	0.4 (0.2, 0.8)	0.2 (0.0, 0.6)	12.3 (10.8, 13.8)
**Age group (yr)**				
15-29	0.2 (0, 1.3)	0	0	0.4 (0.1, 1.2)
30-44	1.5 (0.7, 3.0)	0	0.2 (0, 1.1)	2.3 (1.3, 4.1)
45-59	12.4 (9.5, 16.0)	0.3 (0, 2.0)	0.2 (0, 1.7)	13.1 (10.1, 16.8)
≥ 60	37.7 (33.6, 42.0)	1.4 (0.5, 3.5)	0.5 (0.1, 2.2)	39.8 (35.6, 44.1)
**Area of residence**				
Urban	7.7 (6.4, 9.1)	0.1 (0, 0.6)	0.1 (0, 0.9)	8.0 (6.7, 9.6)
Rural	8.3 (7.3, 9.5)	0.2 (0.1, 0.6)	0.2 (0.1, 0.5)	9.2 (8.1, 10.5)
**Region**				
Central	7.2 (5.9, 8.8)	0.1 (0, 0.7)	0.1 (0, 0.9)	7.4 (6.2, 9.0)
Northeast	10.1 (8.5, 11.9)	0.1 (0, 0.4)	0.2 (0, 1.0)	10.8 (8.9, 13.0)
North	7.8 (6.0, 10.0)	0.6 (0.2, 1.7)	0.2 (0, 1.3)	8.9 (6.8, 11.3)
South	6.4 (4.7, 8.7)	0.2 (0, 0.9)	0	8.1 (6.5, 10.0)
Bangkok	6.2 (4.5, 8.4)	0	0	6.2 (4.5, 8.4)

Age-standardized CKD prevalence stage 3 to 5 estimated for Thai adults aged ≥15 years was 8.9%. The prevalence by disease stages were as follows: stage 3, 8.1%; stage 4, 0.2%, and stage 5, 0.2%. By age group, CKD prevalence increased with advancing age as follows: aged < 29 years (0.4%), 30-44 years (2.3%), 45-59 years (13.1%), and aged ≥60 years (39.8%).

CKD prevalence was slightly higher among persons in rural areas than those in urban areas (9.2% vs 8.0%, *P *< 0.05). CKD prevalence was greater among persons in Northeast (10.8%) than those in other regions (north 8.9%, south 8.1%, Bangkok 6.2%, all *P *< 0.05).

### Characteristics of those with CKD (Table 3)

**Table 3:** Characteristics of CKD based on the MDRD (× 1.233) formula

Characteristics	No CKD (n = 2971)	CKD (stage 3-5) (n = 146)	*P *value
Age, yr (SE)	32.3 (0.4)	56.8 (1.2)	< 0.001
Men, % (SE)	50.1 (1.5)	30.4 (3.4)	< 0.001
*GFR, mL/min/1.73 m^2 ^(SE)	80.8 (0.3)	47.2 (2.5)	< 0.001
*Systolic blood pressure, mmHg (SE)	118.0 (0.6)	129.7 (2.0)	< 0.001
*Hypertension, % (SE)	21.8 (1.3)	49.9 (9.8)	< 0.05
*BMI (SE)	23.1 (0.1)	24.4 (0.7)	0.07
*Cholesterol, mg/dL (SE)	192.4 (1.7)	223.5 (13.1)	< 0.05
*Smoking, % (SE)	25.4 (1.0)	26.0 (8.3)	0.94
*Diabetes, % (SE)	6.5 (1.0)	14.6 (4.2)	0.06

*Age standardized

Those with CKD (GFR < 60 mL/min) had a mean GFR of 47.2 mL/min while those with GFR ≥60 mL/min had a mean of 80.8 mL/min (*P *< 0.001).

The mean systolic blood pressure was higher in CKD than those without CKD (129.7 mmHg vs 118.0 mmHg, *P *< 0.001).

The proportion of hypertension was higher in those with CKD than those without CKD (49.9% vs 21.8%, *P *< 0.05).

Those with CKD also had higher prevalence of diabetes (14.6% vs 6.5%, *P *= 0.06), a higher level of cholesterol (223.5 mg/dL vs 192.4 mg/dL, *P *< 0.05) and greater BMI (24.4 kg/m^2 ^vs 23.1 kg/m^2^, *P *= 0.07) compared to those without CKD.

### Factors associated with CKD

Table 4 shows factors that were associated with CKD in logistic regression model. After controlling for sex, hypertension, diabetes, smoking and BMI, age was strongly associated with CKD, the odds ratio of CKD was 3.0 times for an increase of 10 years of age. CKD was also strongly associated with diabetes (OR, 1.9; 95%CI, 1.0, 3.5), hypertension (OR, 1.6; 95%CI, 1.1, 2.5), BMI (OR, 1.2 for an increase in 1SD).

**Table 4:** Adjusted odds ratios (OR) associated with CKD based on MDRD (× 1.233)

	Adjusted OR	95% confidence interval
Age per 10 year	3.0	2.6, 3.4
Sex (female as reference)	0.3	0.2, 0.5
Diabetes	1.9	1.0, 3.5
Hypertension	1.6	1.1, 2.5
Smoking	1.2	0.6, 2.3
BMI per SD unit	1.2	1.0, 1.5
Total cholesterol per SD unit	1.1	0.8, 1.4
Rural (Urban as reference)	0.9	0.7, 1.3
Region		
Central	1.0	1.0
Northeast	2.1	1.3, 3.3
North	1.7	1.0, 2.8
South	0.8	0.5, 1.3
Bangkok	0.8	0.4, 1.4

(SD of BMI = 4.41 kg/m^2^, SD of cholesterol = 46.76 mg/dL)

Those who reside in northeast were also had a higher risk of CKD compared to those from the central region (OR, 2.1; 95%CI, 1.3, 3.3).

## Discussion

In this study, although we could report only CKD from stage 3 to 5 of 8.9%, the information found is still a useful baseline for further surveillance of situation of CKD in Thailand Early detection is the most important factor needed to tackle CKD, as management could be applied to individual with impaired kidney function to reduce the GFR declination and improve cardioprotection [13]. In order to measure the renal function in this study, we use the MDRD modified glomerular filtration rate estimating equation for Chinese patients with CKD because this formula was provided especially for the Asian population [14]. MDRD has been reported to be more accurate in provide more acceptable estimation of GFR than GC equation in patients with GFR < 60 min/1.73 m^2 ^[15]. The authors chose this equation for improvement in bias, precision and accuracy when compared with the original MDRD equation. Studies in Asian population, specifically in Japanese and Chinese population have reported that the original MDRD formula underestimate of GRF in their population [16,17].

The percentage of the CKD prevalence in Thai adults was previously reported in Royal Thai Air Force (RTAF) personnel who were routinely checked in January 2002 to 2003 [18]. This report included personnel 82 percent men with mean age of 45.7 ± 8 years and was different from our study which included population of both sex from 18 years to over 60 years. The prevalence of CKD in RTAF personnel from stage 3, 4, 5 was 2.9, 0.1, and 0.06% respectively with an overall prevalence from stage 1 to 5 of 4.6% using the MDRD equation and 9.1% using the Cockroft and Gault formula. Another report of CKD prevalence [2] in Thai employees of Electric Generation Authority of Thailand, aged 35-55 years, showed prevalence of CKD stage 3, 4, 5 of 6.4, 0.2, and 0.2% in 1997 to which the author noted this prevalence of CKD was near the same figure as in USA, Singapore, Taiwan and Japan [19-21]. Compared to other developing countries, the prevalence was much lower than that reported in Africa such as Republic of Congo, where the prevalence were 18% in stage 3, 2% in stage 4 and 6% in stage 5 [22]. Recently, there was also an interesting study showing a high prevalence of CKD in Thai population age 35 years and above which was estimated to be about 20% using the Cockroft-Gault formula and about 13% from the MDRD formula [23]. The estimates are higher than that of the present study due to the older age groups and the use of origin formula without adjustment for Asian population. An additional analysis with the original formula in our data provided a relatively high prevalence similar to results of that study [23]; however, we believe that the estimated prevalence derived from the modified formula is appropriate.

This study had some limitations. Firstly, we did not measure proteinuria so that we cannot estimate the early stage of CKD. Secondly, the creatinine measurement was based on Jaffe method which was available at time of the study, current method of IDMS is not available. Thirdly, MDRD equation was based on the original with coefficient correction for Chinese population which might be not appropriate for Thai, as the formula specific for Thai population is not yet available. Lastly, as MDRD systematically underestimates GFR in healthy subject, CKD prevalence tends to be overestimated in epidemiological studies [24,25]. The incidence of ESRD among universal health-care coverage in Thailand has been estimated to be between 101 and 304 per million population (p.m.p.) and although this range quite broad it is comparable to registry-based date from Australia (90-100 p.m.p), Europe (85-160 p.m.p) and the United states (336 p.m.p). Factors that are associated with the high prevalence of CKD in the north and north eastern of Thailand should be urgently sought out. Other risk factors of CKD in this study were similar to other reports and included older age, diabetes mellitus, hypertension, and elevated BMI. The prevalence of CKD raises the possibility of a large burden of RRT over the coming years and has profound public health implications. The findings of this study may offer a potential opportunity for earlier therapeutic interventions to reduce the rate of disease progression and incidence of ESRD.

## Conclusion

CKD was common in Thai adults especially in the north and northeast of Thailand. Effective prevention strategies should be urgently sought and provided.

## Competing interests

The authors declare that they have no competing interests.

## Authors' contributions

All authors contributed substantially to the development of the manuscript, and have read and approved the final paper.

## Pre-publication history

The pre-publication history for this paper can be accessed here:

http://www.biomedcentral.com/1471-2369/10/35/prepub
